# Promoting Help-seeking using E-technology for ADolescents with mental health problems: study protocol for a randomized controlled trial within the ProHEAD Consortium

**DOI:** 10.1186/s13063-018-3157-7

**Published:** 2019-01-31

**Authors:** Michael Kaess, Sabrina Ritter, Sophia Lustig, Stephanie Bauer, Katja Becker, Heike Eschenbeck, Markus Moessner, Christine Rummel-Kluge, Hans-Joachim Salize, Rainer Thomasius, Franz Resch, Julian Koenig, the ProHEAD Consortium, Michael Kaess, Stephanie Bauer, Rainer Thomasius, Christine Rummel-Kluge, Heike Eschenbeck, Hans-Joachim Salize, Katja Becker, Katja Bertsch, Sally Bilic, Romuald Brunner, Johannes Feldhege, Christina Gallinat, Sabine C. Herpertz, Julian Koenig, Sophia Lustig, Markus Moessner, Fikret Özer, Peter Parzer, Franz Resch, Sabrina Ritter, Jens Spinner, Silke Diestelkamp, Kristina Wille, Sabrina Baldofski, Elisabeth Kohls, Lina-Jolien Peter, Vera Gillé, Hanna Hofmann, Laya Lehner, Elke Voss, Jens Pfeiffer, Alisa Samel

**Affiliations:** 1 Section for Translational Psychobiology in Child and Adolescent Psychiatry, Clinic of Child and Adolescent Psychiatry, Centre of Psychosocial Medicine, University of Heidelberg, Blumenstr. 8, 69115 Heidelberg, Germany; 2 University Hospital of Child and Adolescent Psychiatry and Psychotherapy, University of Bern, Stöckli, Bolligenstrasse 141c, 3000 Bern 60, Switzerland; 3 Center for Psychotherapy Research, University Hospital Heidelberg, Bergheimerstr 54, 69115 Heidelberg, Germany; 4 Department of Child and Adolescent Psychiatry, Psychosomatics and Psychotherapy, Philipps-University of Marburg, Hans-Sachs-Str. 6, 35039 Marburg, Germany; 5 Marburg Center for Mind, Brain and Behavior (MCMBB), Philipps-University of Marburg, 35043 Marburg, Germany; 6 Department of Psychology, University of Education Schwäbisch Gmünd, Oberbettringer Str. 200, 73525 Schwäbisch Gmünd, Germany; 7 Department of Psychiatry and Psychotherapy, University Leipzig, Semmelweisstraße 10, 04103 Leipzig, Germany; 8 Mental Health Services Research Group, Central Institute of Mental Health, Medical Faculty Mannheim/Heidelberg University, J5, 68159 Mannheim, Germany; 9 German Center for Addiction Research in Childhood and Adolescence, University Hospital Hamburg-Eppendorf, Martinistr 52, W29, 20246 Hamburg, Germany

**Keywords:** Help-seeking, Mental health, Internet, Adolescents, Randomized controlled trial, ProHEAD

## Abstract

**Background:**

The highest incidence and prevalence of mental health problems across the lifespan as well as the first onset of most long-term mental health conditions are reported for youths between 14 and 25 years of age. At the same time, only 25% of adolescents with mental health problems receive professional treatment. One explanation for poor treatment access in youths is their low help-seeking behavior. Barriers that can keep children and adolescents (C&A) from seeking professional help include a lack of perceived need, structural barriers, or stigma. Interventions based on e-technology might present an effective approach, overcoming these barriers by reducing stigma and providing low-threshold access with enhanced reach, ultimately facilitating help-seeking for mental health problems among youths.

**Methods:**

The study is designed as a multi-center, randomized controlled trial. In total, an estimated number of *n* = 1,500 C&A with mental health problems, drawn from a school-based sample of *n* = 15,000 pupils attending school grades 6 to 13 (≥ 12 years of age), recruited in five regions of Germany, will be randomized either to an intervention (ProHEAD online) or a control condition. C&A in the intervention group will receive online access to tailored information and individual advice on where to seek professional help for their specific needs close to their place of living, case reports of and interaction with peers, as well as the opportunity for online and telephone counseling. C&A in the control intervention will receive a recommendation to seek help and online information on where to find professional help. All participants will be asked to complete questionnaires concerning their help-seeking behavior at baseline, during the intervention (monitoring), and also at a 1 and 2 year follow-up. The primary endpoint is the number of C&A seeking conventional face-to-face professional help in the real-world setting within 1 year after their initial screening.

**Discussion:**

The trial will investigate if an Internet-based intervention can increase professional help-seeking in C&A with mental health problems. With its randomized controlled design and large-scale school-based sampling, the study aims to overcome the shortages of previous research. The intervention has the potential to narrow the treatment gap in C&A and to ultimately improve the mental health care system.

**Trial registration:**

German Clinical Trials Register, DRKS00014685. Registered on 7 July, 2018.

**Electronic supplementary material:**

The online version of this article (10.1186/s13063-018-3157-7) contains supplementary material, which is available to authorized users.

## Background

Children and adolescents (C&A) are frequently affected by psychiatric illness and mental health problems. Recent population-based studies report a 50% incidence of mental health problems in the age group between 12 and 25 years, and a 12-month prevalence of 40% for those between 13 and 18 years of age [1]. Mental health problems in this age group are associated with a high risk of persistence and serious functional impairment, emphasizing their long-term impact [2]. A recent review reported that neuropsychiatric disorders are the most common causes of disability (45%) in individuals between 10 to 24 years of age [3]. In sum, the highest incidence and prevalence of mental health problems across the lifespan and the first onset of most long-term mental health conditions are reported for youth between 14 and 25 years of age [4].

At the same time, and most alarmingly, youths clearly show the worst service access [4]. There is evidence that only 20–40% of adolescents with mental health problems are actually detected by health services and only 25% receive appropriate professional treatment [5]. This problem has been repeatedly confirmed for a variety of highly prevalent mental health problems such as depression [6], eating disorders [7], and substance misuse [8]. A representative study throughout Europe that included a school-based screening of *n* = 13,070 C&A (13–17 years of age) showed that at least 12.5% were in need of mental health care. However, less than one third took the offer of receiving direct professional help [9], illustrating very low help-seeking behavior among European adolescents at risk. These data fit those of previous studies concluding that C&A with mental health problems often do not receive treatment due to low help-seeking behavior [10, 11]. This is highly worrying, as the group of older C&A (aged 12–17) can be seen as the most important target group for early detection of individuals with mental health problems. Early detection increases the chance of early treatment, thus diminishing the risk of recurrence and/or serious residual damage, and thereby providing an opportunity to improve psychosocial outcomes and reduce health economic costs [12, 13].

Several barriers have been identified that potentially keep C&A from accessing mental health services [14]. A lack of perceived need for services, preference for self-management, fear of hospitalization, a lack of service availability within a reasonable time, lack of information, and structural factors (e.g., distance, finances) have been identified as key barriers to care [15]. Key components of C&A-friendly services (according to the World Health Organization) are availability, easy accessibility, equitability (e.g., being non-judgmental; open for all young people regardless of gender, culture, marital status, socioeconomic status, etc.), acceptability (e.g., having clear policies about confidential and patient-centered care), and appropriateness (e.g., staffed by skilled clinicians) [16].

By combining these features, interventions based on e-technology might present an effective approach to overcome barriers of help-seeking and to facilitate access to conventional care. Over the past decade, technology has played an increasingly bigger role in the delivery of psychosocial and psychotherapeutic interventions (“e-mental health”). E-mental health and Internet-based interventions have the advantage of easy, low-threshold access, enhanced reach, including traditionally underserved populations, relatively low cost, and time efficiency. In addition, technology allows for providing flexible interventions that are tailored to the individual needs and preferences of participants. Across the spectrum of mental health problems, growing evidence points to the potential of e-interventions for prevention, self-help treatment, counseling, and relapse prevention, and also as an adjunct to conventional psychotherapy using various forms of media and technology [17, 18]. It is assumed that Internet-based interventions may improve mental health literacy and contribute to a de-stigmatization of mental illness, thus promoting help-seeking attitudes, intentions, and ultimately behaviors [19]. However, only a few studies attempted to utilize Internet-based interventions to promote mental help-seeking. A recent review identified 18 studies, all with major methodological limitations (i.e., small sample sizes, lack of control group, no follow-up, and failure to assess behavioral outcomes) [20]. Furthermore, existing Internet-based interventions mostly address one particular health condition (mainly depression or anxiety), rather than providing different modules for a broad range of mental health problems prevalent in C&A all integrated in one superordinate program. Promoting Help-seeking using E-technology for ADolescents (ProHEAD) is such a superordinate program, which covers mental health problems (i.e., conduct problems, hyperactivity/inattention, peer relationship problems, emotional problems, eating disorders, addiction, and suicidality) not jointly covered by other programs. Finally, previous research is limited by the fact that the interventions aiming to improve help-seeking have almost exclusively consisted of one-time, fully automated tools (mostly psychoeducational content), not giving consideration to the heterogeneous and complex pathways to care [15]. By specifically addressing these methodological issues, an intervention based on e-technology bears great potential to conquer various barriers of help-seeking in C&A, to facilitate service access, and to finally contribute to relieve the burden of mental disease in youths.

## Objectives

The aim of the present study is to develop, implement, and evaluate an Internet-based program to promote help-seeking in C&A with mental health problems (i.e., scoring above critical thresholds in validated self-report questionnaires on diverse emotional and behavioral problems) across all disorders in a randomized controlled trial (RCT). The program will make use of this age group’s familiarity with the Internet to provide a low-threshold access to mental health assistance.

## Hypotheses

The primary hypothesis is that a greater proportion of C&A with mental health problems who are randomized (intention-to-treat) to help-seeking assistance through an individualized online based intervention are more likely to actually utilize professional (formal) face-to-face mental health care from a child and adolescent psychiatrist or psychotherapist within 1 year (primary endpoint), compared to a control group receiving information only. Secondary hypotheses are that C&A allocated to the intervention group will score more favorably on measures of mental health problems, health-risk behaviors, and quality of life in the respective follow-up assessments, compared to the control group. Additionally, health economic analyses will be conducted to assess economic aspects of the newly developed intervention.

## Methods/design

### Setting and recruitment

The trial is part of a multi-center consortium situated at six study sites across Germany and led by the managing site at the Clinic of Child and Adolescent Psychiatry at the University Hospital of Heidelberg (for details on the consortium, see the Editorial “*Promoting Help-seeking using E-Technology for ADolescents: The ProHEAD Consortium*”). The study protocol was approved by the Ethics Committee of the Medical Faculty at the University of Heidelberg.

A school-based sample of *n* = 15,000 C&A in grades 6–13 (≥ 12 years of age) will be recruited at five regions in Germany (Hamburg, Heidelberg, Leipzig, Marburg, Schwäbisch Gmünd). Permission to contact schools within the regional districts of all five recruiting sites will be requested from federal authorities. A complete list of schools in regional districts will be acquired. Schools are randomly selected for each school type separately to ensure a random selection of schools that ultimately represents the distribution of school types within the recruitment area. The school list is stratified by regional district^1^ and school-type.^2^ Within these strata the order of schools is random. Regarding the intervention, individual-level randomization is performed on all eligible participants to ensure timely allocation to one treatment arm. Schools of the respective school types will be contacted and informed about the possibility of participating in the trial in random order, until the prospected sample size by site is reached. Eligible C&A (≥ 12 years of age, sufficient German language skills, access to the Internet) and their legal guardians are asked to provide written informed consent and participate in a school-based screening covering various forms of mental health problems (detailed subsequently).^3^ Study personnel will check back on the return of the written informed consent sheets a couple of weeks after an informative class meeting and the distribution of study information materials. On the day of the assessment, C&A will further receive an emergency contact card, detailing procedures in the case of emergency or urgent request for professional consultation. School-based assessments will be repeated after 12 and 24 months.

Based on the screening results, each participant will be allocated to one of the five Internet-based trials (general mental health problems [this RCT]; eating disorder symptoms [21]; risky alcohol use [22]; depressive symptoms [23]; no mental health problems [24]). C&A meeting inclusion criteria for more than one RCT will be randomly allocated to one of the RCTs. Criteria for the allocation of participants to the five individual ProHEAD RCTs are based on the latest scientific evidence from epidemiological studies. However, this is the first time that the overall algorithm is applied on a consortium-wide basis simultaneously screening for various mental health problems. Therefore, a preliminary data analysis will be conducted following completion of 10% of the screening assessments (*n* = 1,500) in order to determine the actual allocation ratio to the five ProHEAD trials and to adjust the screening algorithm if necessary.

### Inclusion and exclusion criteria

C&A from the school-based sample (≥ 12 years of age, sufficient German language skills, access to the Internet) are included in the present clinical trial if they endorse any form of mental health problems, including serious suicide thoughts or attempts in the past 2 weeks, a score above 19 points on the Strengths and Difficulties Questionnaire [25] total score, or a score above the defined thresholds for one of its sub-scales: *emotional symptoms* (scores > 6), *conduct problems* (scores > 4), *hyperactivity/inattention* (scores > 6), or *peer relationship problems* (scores > 5). Further, C&A will be included if they report the following: body mass index (BMI) < 5th percentile (adjusted for age and gender) AND concurrent fear of weight gain OR daily binge eating OR daily vomiting OR current alcohol use disorder [26] OR a score above 9 on the Patient Health Questionnaire-9 modified for adolescents (PHQ-A) [27]. C&A scoring below all clinical cut-offs are allocated to other trials nested within the ProHEAD Consortium. C&A not providing written informed consent (including parental consent) will be excluded.

### Randomization and blinding

**Fig. 1:**
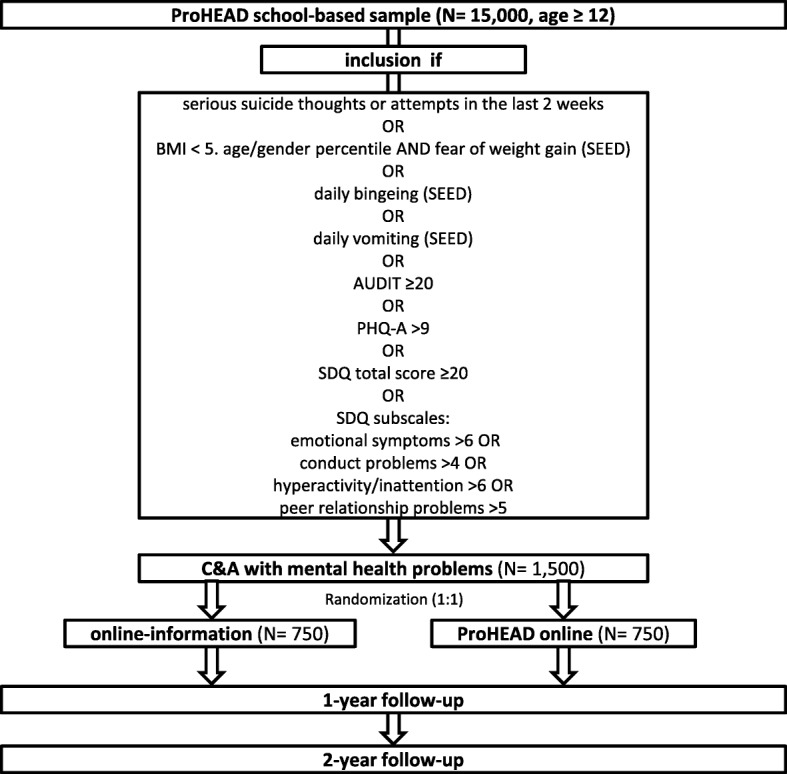
ProHEAD online trial flow. *SEED* Short Evaluation of Eating Disorders, *AUDIT* Alcohol Use Disorders Identification Test, *PHQ-A* Patient Health Questionnaire-9 modified for adolescents, *SDQ* Strengths and Difficulties Questionnaire, *C&A* Children and adolescents

For the current trial, eligible C&A will be randomized to one of the two treatment arms based on a permuted block design (Fig. 1). Randomization will be automatically performed via a predefined algorithm after the school-based screening on an individual subject level to ensure timely allocation and allocation concealment. Participating C&A will receive an email with a link to activate their personal account in the allocated group. Participants cannot be blinded due to the different natures of the interventions. Blinding of the researchers is non-applicable. The data analysts will be blinded to group allocation (dummy coded) when conducting the statistical analyses.

### Sample size

Based on the expected sample size to be allocated by randomization (*n* = 1,500), a power analysis for the trial revealed that the study is powered to detect small effects (1.13 ratio, i.e., 13% differences in help-seeking between groups, alpha = 5%), assuming a critical χ^2^ = 34.55. All tests will be two-sided.

### Data assessment

In addition to sociodemographic information (i.e., migration status, socioeconomic status), the school-based screening will cover screening instruments for a broad range of mental health problems. All measures have previously been used in adolescent samples [28–30]. Three self-report instruments will be used to cover help-seeking intentions, actual help-seeking behavior, and attitudes toward help-seeking. The General Help-Seeking Questionnaire (GHSQ [28]) is a self-report measure of help-seeking intentions. Help-seeking intentions for selected mental health problems are assessed on an 8-point scale ranging from 1 (extremely unlikely) to 8 (extremely likely). The Actual Help-Seeking Questionnaire (AHSQ) [31] assesses actual help-seeking behavior by listing potential sources of help and measuring whether or not help has been sought from the respective sources within a specified time period (in the last 12 months; more than 12 months ago). It comprises three sub-scales: whether or not informal help has been sought, whether or not formal help has been sought, and whether no help has been sought. Further, the Inventory of Attitudes Toward Seeking Mental Health Services (IASMHS) [32] will be used. The IASMHS is a 24-item scale and has three internally consistent factors: *psychological openness*, *help-seeking propensity*, and *indifference to stigma*. To assess barriers of help-seeking, 12 items were generated based on a literature review of help-seeking barriers and compared with the validated instrument Barriers to Adolescents Seeking Help Scale (BASH-B [33]). Further, items from the Questionnaire on Social Distance [34], assessing stigma towards peers affected by mental health problems, will be implemented in the screening. Health care utilization of study subjects will be collected by the *Mannheimer Modul Ressourcenverbrauch* (MRV), a scale that lists all possible health care services for a given study sample or risk group and reports the frequency of usage (visits, drug intake, hospital days, etc.) over a given period of time [35]. Similar scales are applied in international cost studies [36]. The MRV was modified and pretested for its use in an adolescent population.

**Fig. 2:**
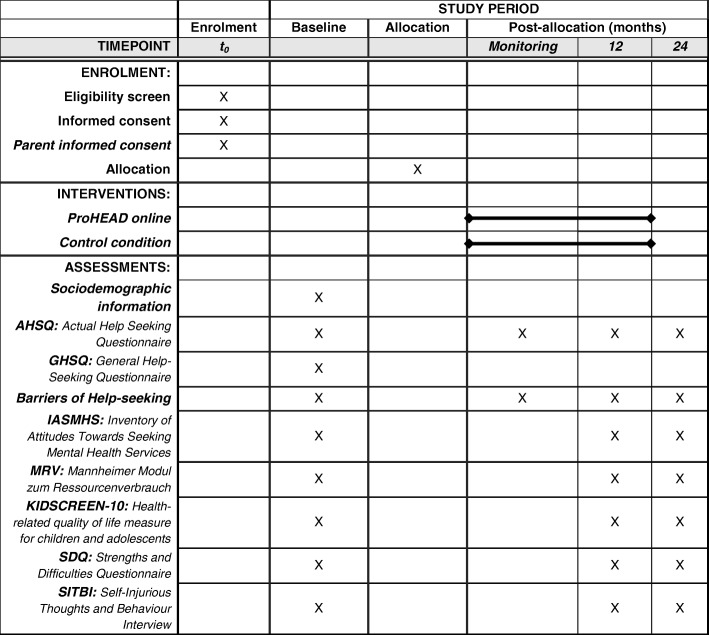
Schedule of enrollment, interventions, and assessments. *Note:* Monitoring 1: 7 days after registration, Monitoring 2–7: every 14 days for the following 10 weeks, Monitoring 8–14: every 28 days for the following 32 weeks, Monitoring 15: 301 days after registration

Figure 2 displays an overview of enrollment, interventions, and measures used as well as the corresponding time of assessment. In Additional file 1 a populated Standard Protocol Items: Recommendations for Interventional Trials (SPIRIT) checklist is provided. Additional file 2 provides the World Health Organization Trial Registration Data Set.

Participants receive no direct financial compensation for participating in the school-based assessments. Among all participating C&A, a lottery will be conducted, awarding online gift vouchers (20€ in value) to 5% of the participants.

### Intervention

The intervention is individually delivered via the Internet. All C&A enrolled in this trial receive log-in information to an Internet-based platform that requires secured log-in. The web-based platform contains public content and a personal area with user-specific information and, depending on the group allocation, access to intervention modules. For participants in the control condition, the platform only contains information on the individual results of the school-based assessment and advice to seek professional counseling within the mental health care system. Moreover, the control group is granted access to addresses and names of local mental health care professionals and prompted by a static website encouraging them to seek help at the respective institutions. C&A in the control condition will only be contacted once and are not reminded or followed up outside of the school-based follow-up assessments. The design is justified by testing ProHEAD online against a real-world condition.

C&A allocated to ProHEAD online (intervention group) will receive their individual screening results, individual advice to seek professional help, and contact information for local mental health care professionals. Moreover, the intervention group is granted access to three additional modules: the *Information and Education Module*, the *Motivation and Guided Referral Module*, and the *Monitoring Module*.

#### Information and Education Module

C&A with mental health problems are provided with symptom- and gender-adequate information about mental health problems, personalized to their individual screening profile as assessed within the school-based screening. The module aims at providing tailored psychoeducation to affected C&A in order to reduce the stigma associated with psychopathology and increase mental health literacy. Information on the causes and consequences of mental health problems as well as their prevalence and the possibilities for intervention are provided in a youth-adequate manner. Information was collected and summarized by staff with different occupational backgrounds in consensus with C&A experts in Germany as well as representatives from societies representing patients’ interest. The information was reviewed by a specially invited focus group consisting of C&A between 12 and 14 years with mental health problems. To achieve a broad, comprehensive, and complete *Information and Education Module*, female and male C&A with different diagnoses were invited. These discussions led to revisions of content that weres ultimately implemented in the respective modules.

C&A can access information on all different forms and facets of mental health problems, but specific topics and chapters are highlighted based on their individual needs according to their screening profile in their personal area.

#### Motivation and Guided Referral Module

The *Motivation and Guided Referral Module* provides an important transit between the *Information and Education Module* and actual referral to real-world services. ProHEAD online aims to motivate C&A with mental health problems to seek professional diagnostic assessments and potentially therapeutic help within the conventional face-to-face mental health care systems in manifold ways. Case examples from peers are provided to reduce the stigma and prejudices about seeking and receiving help for mental health problems in order to promote a sense of community among C&A with mental health problems. Further, ProHEAD online offers case management, enabling C&A to chat with trained case managers at youth-adequate times (4 to 10 p.m.) through the ProHEAD online web portal, providing individual guidance and support. C&A are provided with addresses of local authorities and institutions and are offered advice in approaching mental health care services according to their individual needs and preferences. Case managers closely guide and counsel C&A on their way, requesting scheduled updates on individuals’ progress and outcomes. The case managers re-contact C&A via email within defined time frames of disengagement with the web-based platform, to improve binding and gain regular updates on their individual status.

#### Monitoring Module

A monitoring system allows for the gathering of information regarding the help-seeking behavior of C&A in the intervention group. This will enable the case managers to tailor the intervention to a participants´ individual status, situation, and needs, allowing for adequate and tailored support of C&A. We distinguish automatized monitoring from individual monitoring. Automatized monitoring occurs based on user-website interaction in terms of 15 regular online assessments (Monitoring 1: 7 days after registration; Monitoring 2–7: every 14 days for the following 10 weeks; Monitoring 8–14: every 28 days for the following 32 weeks; Monitoring 15: 301 days after registration). As part of the automatized monitoring, C&A receive emails inviting them to complete a short questionnaire and motivating them to stay engaged with the platform. Automatized monitoring ends if C&A report successful help-seeking within the professional mental health care system. Alongside automated prompts, individual monitoring is realized via a team of case managers; each case manager tracks the progress of particular C&A. The case managers have different occupational backgrounds (e.g., psychologist, health education professional) and are trained in motivational interviewing [37] and all study-specific procedures (i.e., administration of the program). Individual monitoring includes individual communication (chat, phone, and email) regarding the progress and status on a regular basis. After registration participants will have access for up to 10 months. The dose of the intervention is primarily determined by the participating C&A. C&A are free to log in to the web-based platform according to their needs and interest. The system will send automated reminders to C&A who stay absent of the platform and have not made efforts to seek conventional face-to-face professional help.

### Outcomes

The primary endpoint of the study is the 1-year follow-up, where all participants will be assessed on mental health problems and help-seeking intentions and behaviors, as well as actual help-seeking within the past year (primary outcome) in a second school-based assessment. A long-term follow-up will take place 2 years after the initial screening. C&A not participating at the school-based follow-up assessment will receive individual notices via email including a link to complete the assessment outside of the school environment if possible. All other medications and treatments used by participating C&A are permitted and will be assessed at the follow-up school-based screenings.

### Statistical analysis

The main hypothesis is that a greater proportion of those C&A with mental health problems who are randomized to ProHEAD online (intention-to-treat) will present themselves within the conventional professional mental health care system after 1 year compared to C&A in the control condition. This will be addressed using chi-squared tests with Fisher’s exact *F* to adjust for zero inflation of cell distribution sizes (C&A who utilized professional help versus C&A who did not utilize professional help) on group differences. In secondary analyses, predictors of help-seeking behavior (i.e., sex, age, utilization of the online intervention, and symptom severity at initial school-based screening) will be assessed using multinomial logistic regression analyses. Engagement time per participant (minutes per day, days per month) and content of engagement will be tracked to get a reliable estimate of the utilization of the platform that is of interest for analyses of the dose-response relationship. Missing data and subjects withdrawn from the trial will be handled using an intention-to-treat approach. All subjects randomized will be considered in the analyses. In the case of drop-outs or missing data, it will be assumed that these C&A did not present themselves within the mental health care system to provide a conservative estimate of the true effect. Potential class and school effects will be tested and adjusted for if necessary.

In addition, a cost-effectiveness analysis, including the calculation of the incremental cost-effectiveness ratio (ICER), will be conducted. To provide information on cost per quality-adjusted life year (QALY), cost-utility analyses will be used.

### Data monitoring and safety

All data will be collected via central servers that are used for both the school-based assessments and the intervention conducted via the ProHEAD platform (www.prohead.de). All study data will be stored under a code, ensuring complete pseudonymization. Computerized assessments guarantee the highest level of data integrity and quality; i.e., missing data will be minimized, and false data entry will be prevented. Online access allows for continuous monitoring of data collection, documentation of access logs, and traceability of all entered data (user and timestamp) as well as restoration of all previous states. A Distributed Replicated Block Device (DRBD)-based cluster will provide synchronous replication of all data during data entry on two separate servers, as well as highest availability. In addition, full and incremental backups will be conducted following a predefined backup plan.

Data will be handled in accordance with German legal regulations concerning data protection and data security (Landesdatenschutzgesetz Baden-Württemberg and Bundesdatenschutzgesetz) as well as European Union (EU) General Data Protection Regulation. Data storage and transfer will be encrypted. Access to the data will be strictly limited to authorized persons and will be password-protected. All servers are located at the University Hospital Heidelberg. Data will be stored for at least 10 years at the primary research institution. The data will be accessible for project partners and their respective statistical experts.

Monitoring will be done according to the Guidelines for Good Clinical Practice (ICH-GCP). The Coordination Center for Clinical Trials (KKS) Heidelberg will oversee the study procedures at all five recruiting centers. In particular, the recruitment of schools and the students within these schools will be monitored in order to obtain adherence to the study manual and documentation guidelines and to ensure equivalent procedures at all sites.

An independent Data and Safety Monitoring Committee (DSMC) is formed by PD Dr. phil. Annette Conzelmann (University of Tübingen), Prof. Dr. rer. nat. Kerstin Konrad (University Hospital Aachen), and Prof. Dr. med. Susanne Walitza (University of Zurich). The DSMC will oversee all aspects of data collection, handling, and analysis.

### Stopping rules

Stopping rules for C&A participating in the trial are the reporting of acute suicide plans or suicide attempts while participating in the ProHEAD intervention, as communicated with the case manager. In case of the reporting of acute suicide plans or attempts, special emergency procedures will be put in place that allow immediate contact with the participant in order to assess risks and refer to appropriate care. The case manager will try to get the participant to immediately seek help via an ambulance or the police. If no commitment can be achieved for voluntary help-seeking, the case managers will ascertain the first name and surname of the participant to report the plans to the local police. Further, C&A who withdraw consent to participate in the trial will discontinue participation. There are no discontinuation criteria for the whole trial.

### Ethical issues

The study will be conducted in accordance with the Declaration of Helsinki and the regulations for physicians of the medical association (Landesärztekammer) of Baden-Württemberg in their currently valid version. Study participation is voluntary. A participant can withdraw consent at any time without stating the reason and without any individual disadvantage for subsequent medical care. Study participants and their parents or alternatively persons with parental authority will be informed in writing about the procedures and potential undesirable effects or risks of the study. Their approval will be documented via their signature on the informed consent forms. The Ethics Committee of the Medical Faculty at the University of Heidelberg will be informed in case of severe adverse events or other unintended effects of the trial interventions.

### Dissemination of results

In addition to research publications and conference contributions, the ProHEAD Consortium will take several measures to disseminate the results beyond the scientific community. Information about the project and the availability of the Internet-based interventions and e-mental health tools (after-study stage) will be provided to patients and health care providers as well as to youth organizations and schools. Awareness in the general public will be increased by the ProHEAD website (www.prohead.de) and press campaigns accompanying the development of the project, disseminating its results.

## Discussion

The aim of the trial is to investigate if an Internet-based intervention can increase help-seeking behavior in C&A with mental health problems. Existing empirical research suggests that e-interventions are indeed capable of promoting help-seeking behavior in youths [38, 39]. Most C&A prefer technology-based interventions over a face-to-face in-person contact [40]. Internet-based mental health services offer anonymous help in an unobtrusive but easily accessible way that is modern and age-appropriate. In this manner, C&A in need can be reached who otherwise would not find their way into the health care system, e.g., because of stigma, fear, or structural reasons. However, some interventions based on e-technology previously implemented failed to help participants to ask for professional help [41, 42]. A systematic review analyzed 18 studies investigating the effects of e-interventions on young people’s help-seeking and identified a number of shortcomings in existing studies [20]. According to this review, some trials showed no methodological rigor (e.g., a lack of control group, no follow-up assessments) or included participants with mild mental health problems only. Furthermore, the majority of programs placed an emphasis on information only, did not include interactive modules, and were evaluated in small community-based samples. Using a randomized controlled design on a large-scale sample, the present study aims to overcome these shortcomings. The intervention is suitable for a broad range of C&A affected by different mental health problems with clinical relevance. At the same time, a unique strength of the intervention is the individual mentoring, enabling customized support for each participant. Cooperation with consortium partners all over Germany allows for the recruitment of a representative sample of *n* = 15,000 C&A, of which *n* = 1,500 (10%) are expected to fulfill eligibility criteria for the present trial. The consortium members have long-standing experience in school-based recruitment and mental health assessment of C&A [43–45]. The school-based screening reaches all C&A without self-selection bias and enables an intention-to-treat approach.

Despite the significant advantages of a large scale multi-center study, running a trial in different study centers comes with special demands. There is a risk that single study sites may perform differently in recruitment, placing a requirement for special attention on standardized study implementation. For this purpose, a special training for recruiting staff from all study sites will take place prior to the baseline screening to ensure strict adherence to the study manual. Standardized information material further supports comparable results and reduces study site-specific biases. Participation in the study does not present any obvious risks for C&A. All participants, including those in the control condition, will receive information on where to seek help for mental health problems.

### Implications and future impact

Young people are familiar with the Internet and online programs. They can access them at any time in accordance with their individual needs. Therefore, an online intervention might be the superior way to provide tailored information and low-threshold access to enhance help-seeking for mental health problems among youths.

Especially in this population, interventions are needed, because early detection increases the chance of early treatment. This diminishes the risk of recurrence and/or serious residual damage, thereby providing an opportunity to improve psychosocial outcomes and reduce health economic costs [12, 13].

If the intervention is shown to be effective, the present study has the potential to narrow the treatment gap in C&A and to ultimately improve the mental health care system.

## Trial status

The recruitment of the school-based sample within the ProHEAD Consortium will start in October 2018 with the baseline assessment and last until March 2020.

## Additional files

Additional file 1: SPIRIT 2013 checklist: recommended items to address in a clinical trial protocol and related documents. (DOC 121 kb)

Additional file 2: World Health Organization Trial Registration Data Set. (DOCX 19 kb)

## Abbreviations

AHSQ: Actual Help-Seeking Questionnaire
AUDIT: Alcohol Use Disorders Identification Test
BASH-B: Barriers to Adolescents Seeking Help Scale
C&A: Children and adolescents
DSMC: Data and Safety Monitoring Committee
GHSQ: General Help-Seeking Questionnaire
IASMHS: Inventory of Attitudes Toward Seeking Mental Health Services
ICH-GCP: Guideline for Good Clinical Practice
KIDSCREEN: Health-related quality of life measure for children and adolescents
KKS: Coordination Center for Clinical Trials (Koordinierungszentrum für Klinische Studien)
MRV: Mannheimer Modul Ressourcenverbrauch
PHQ-A: Patient Health Questionnaire-9 modified for adolescents
ProHEAD: Promoting Help-seeking using E-technology for ADolescents
RCT: Randomized controlled trial
SDQ: Strengths and Difficulties Questionnaire
SEED: Short Evaluation of Eating Disorders
SITBI: Self-Injurious Thoughts and Behaviors Interview

## Footnotes

1 Due to the regional specifics of the rural area around the recruitment site Schwäbisch Gmünd, a preselection was made. Only schools within a radius of 30 km were included.

2 School types in Germany: *Gymnasium*, 8 or 9 years of school after 4 years of elementary school, terminating with the general qualification for university entrance; *Realschule*, 6 years of school after 4 years of elementary school, terminating with a secondary-school level certificate; *Hauptschule*, 9 years of elementary school, basis of vocational education; *Mittel-/Oberschule*: secondary school certificate; *Gemeinschaftsschule*/*Gesamtschule/Stadtteilschule* all qualifications are possible (qualification for vocational education, secondary school certificate and general qualification for university).

3 Note: The school-based ProHEAD screening covers a broad range of questionnaires and instruments. Detailed below are the ones most relevant to the present clinical trial. For further details on other instruments, please refer to the respective study protocols published alongside this protocol.

## References

[1] Kessler RC, Petukhova M, Sampson NA, Zaslavsky AM, Wittchen H-U (2012). Twelve-month and lifetime prevalence and lifetime morbid risk of anxiety and mood disorders in the United States: anxiety and mood disorders in the United States. Int J Methods Psychiatr Res.

[2] Kim-Cohen J, Caspi A, Moffitt TE, Harrington H, Milne BJ, Poulton R (2003). Prior juvenile diagnoses in adults with mental disorder: developmental follow-back of a prospective-longitudinal cohort. Arch Gen Psychiatry.

[3] Gore FM, Bloem PJ, Patton GC, Ferguson J, Joseph V, Coffey C (2011). Global burden of disease in young people aged 10–24 years: a systematic analysis. Lancet.

[4] Gibb SJ, Fergusson DM, Horwood LJ (2010). Burden of psychiatric disorder in young adulthood and life outcomes at age 30. Br J Psychiatry.

[5] Sanci L, Lewis D, Patton G (2010). Detecting emotional disorder in young people in primary care. Curr Opin Psychiatry.

[6] Essau CA (2005). Frequency and patterns of mental health services utilization among adolescents with anxiety and depressive disorders. Depress Anxiety.

[7] Hart LM, Granillo MT, Jorm AF, Paxton SJ (2011). Unmet need for treatment in the eating disorders: a systematic review of eating disorder specific treatment seeking among community cases. Clin Psychol Rev.

[8] Cunningham JA, Breslin FC (2004). Only one in three people with alcohol abuse or dependence ever seek treatment. Addict Behav.

[9] Wasserman D, Carli V, Wasserman C, Apter A, Balazs J, Bobes J (2010). Saving and Empowering Young Lives in Europe (SEYLE): a randomized controlled trial. BMC Public Health.

[10] Gould MS, Marrocco FA, Hoagwood K, Kleinman M, Amakawa L, Altschuler E (2009). Service use by at-risk youths after school-based suicide screening. J Am Acad Child Adolesc Psychiatry.

[11] Husky MM, McGuire L, Flynn L, Chrostowski C, Olfson M (2009). Correlates of help-seeking behavior among at-risk adolescents. Child Psychiatry Hum Dev.

[12] Haller DM, Sanci LA, Sawyer SM, Patton GC (2009). The identification of young people’s emotional distress: a study in primary care. Br J Gen Pract.

[13] Patel V, Flisher AJ, Hetrick S, McGorry P (2007). Mental health of young people: a global public-health challenge. Lancet.

[14] Tylee A, Haller DM, Graham T, Churchill R, Sanci LA (2007). Youth-friendly primary-care services: how are we doing and what more needs to be done?. Lancet.

[15] Hom MA, Stanley IH, Joiner TE (2015). Evaluating factors and interventions that influence help-seeking and mental health service utilization among suicidal individuals: a review of the literature. Clin Psychol Rev.

[16] Arnett J (2004). Emerging adulthood: the winding road from the late teens through the twenties.

[17] Emmelkamp PMG, David D, Beckers T, Muris P, Cuijpers P, Lutz W (2014). Advancing psychotherapy and evidence-based psychological interventions. Int J Methods Psychiatr Res.

[18] Andersson G, Cuijpers P, Carlbring P, Riper H, Hedman E (2014). Guided Internet-based vs. face-to-face cognitive behavior therapy for psychiatric and somatic disorders: a systematic review and meta-analysis. World Psychiatry.

[19] Taylor-Rodgers E, Batterham PJ (2014). Evaluation of an online psychoeducation intervention to promote mental health help seeking attitudes and intentions among young adults: randomised controlled trial. J Affect Disord.

[20] Kauer SD, Mangan C, Sanci L (2014). Do online mental health services improve help-seeking for young people? A systematic review. J Med Internet Res.

[21] Bauer S, Bilic S, Reetz C, Oezer F, Becker K, Eschenbeck H, et al. Efficacy and Cost-Effectiveness of Internet-based Selective Eating Disorder Prevention: study protocol for a randomized controlled trial within the ProHEAD Consortium. (submitted). 10.1186/s13063-018-3161-y PMC6354385 30700318

[22] Diestelkamp S, Wartberg L, Kaess M, Bauer S, Rummel-Kluge C, Becker K, et al. Effectiveness of a web-based screening and brief intervention with weekly text-message-initiated individualised prompts for reducing risky alcohol use among teenagers: study protocol of a randomised controlled trial within the ProHEAD Consortium. Trials 2019 20:73 10.1186/s13063-018-3160-z. 10.1186/s13063-018-3160-z PMC6341631 30670102

[23] Baldofski S, Kohls E, Bauer S, Becker K, Bilic S, Eschenbeck H, et al. Efficacy and Cost-Effectiveness of Two Online Interventions for Children and Adolescents at Risk for Depression (E.motion trial): study protocol for a randomized controlled trial within the ProHEAD Consortium. Trials 2019;20:53. 10.1186/s13063-018-3156-8. PMC6334409 30646944

[24] Eschenbeck H, Lehner L, Hofmann H, Bauer S, Becker K, Diestelkamp S, et al. School-Based Mental Health Promotion in Children and Adolescents with StresSOS using Online or Face-to-Face Interventions: study protocol for a randomized controlled trial within the ProHEAD Consortium. Trials 2019;20:64. 10.1186/s13063-018-3159-5. PMC6339406 30658675

[25] Goodman A, Goodman R (2009). Strengths and Difficulties Questionnaire as a dimensional measure of child mental health. J Am Acad Child Adolesc Psychiatry.

[26] Babor TF, Higgins-Biddle JC, Saunders JB, Monteiro MG (2001). The Alcohol Use Disorders Identification Test: guidelines for use in primary care.

[27] Johnson JG, Harris ES, Spitzer RL, Williams JBW (2002). The patient health questionnaire for adolescents: validation of an instrument for the assessment of mental disorders among adolescent primary care patients. J Adolesc Health.

[28] Wilson CJ, Deane FP, Ciarrochi J, Rickwood D. Measuring Help-Seeking Intentions: Properties of the General Help-Seeking Questionnaire. Canadian Journal of Counselling. 2005;39(1):15-28.

[29] Jackson Williams D (2014). Help-seeking among Jamaican adolescents: an examination of individual determinants of psychological help-seeking attitudes. J Black Psychol.

[30] Beals-Erickson SE, Roberts MC (2016). Youth development program participation and changes in help-seeking intentions. J Child Fam Stud.

[31] Rickwood DJ, Braithwaite VA (1994). Social-psychological factors affecting help-seeking for emotional problems. Soc Sci Med.

[32] Munson MR, Floersch JE, Townsend L (2009). Attitudes toward mental health services and illness perceptions among adolescents with mood disorders. Child Adolesc Social Work J.

[33] Kuhl J, Jarkon-Horlick L, Morrissey RF (1997). Measuring barriers to help-seeking behavior in adolescents. J Youth Adolesc.

[34] Angermeyer MC, Matschinger H (1997). Social distance towards the mentally ill: results of representative surveys in the Federal Republic of Germany. Psychol Med.

[35] Voß E, Salize HJ (2016). Health care utilization and cost-effectiveness analyses in prevention studies in the mental health care field. Ment Health Prev.

[36] Salize HJ, Kilian R, Gaebel W, Müller-Spahn F (2010). Gesundheitsökonomie in der Psychiatrie: Konzepte, Methoden, Analysen.

[37] Rollnick S, Miller WR (1995). What is motivational interviewing?. Behav Cogn Psychother.

[38] Aardoom JJ, Dingemans AE, Boogaard LH, Van Furth EF (2014). Internet and patient empowerment in individuals with symptoms of an eating disorder: a cross-sectional investigation of a pro-recovery focused e-community. Eat Behav.

[39] King CA, Eisenberg D, Zheng K, Czyz E, Kramer A, Horwitz A (2015). Online suicide risk screening and intervention with college students: a pilot randomized controlled trial. J Consult Clin Psychol.

[40] Ranney ML, Choo EK, Spirito A, Mello MJ (2013). Adolescents’ preference for technology-based emergency department behavioral interventions: does it depend on risky behaviors?. Pediatr Emerg Care.

[41] Costin DL, Mackinnon AJ, Griffiths KM, Batterham PJ, Bennett AJ, Bennett K (2009). Health e-cards as a means of encouraging help seeking for depression among young adults: randomized controlled trial. J Med Internet Res.

[42] Gulliver A, Griffiths KM, Christensen H, Mackinnon A, Calear AL, Parsons A (2012). Internet-based interventions to promote mental health help-seeking in elite athletes: an exploratory randomized controlled trial. J Med Internet Res.

[43] Kaess M, Brunner R, Parzer P, Carli V, Apter A, Balazs JA (2014). Risk-behaviour screening for identifying adolescents with mental health problems in Europe. Eur Child Adolesc Psychiatry.

[44] Kaess M, Parzer P, Haffner J, Steen R, Roos J, Klett M (2011). Explaining gender differences in non-fatal suicidal behaviour among adolescents: a population-based study. BMC Public Health.

[45] Kaess M, Parzer P, Brunner R, Koenig J, Durkee T, Carli V (2016). Pathological Internet use is on the rise among European adolescents. J Adolesc Health.

