# Supplementary material for: Promoting Help-seeking using E-technology for ADolescents with mental health problems: study protocol for a randomized controlled trial within the ProHEAD Consortium
Source: Trials. 2019 Jan 31;20:94. doi: 10.1186/s13063-018-3157-7 (PMC6357507; doi:10.1186/s13063-018-3157-7)
Supplement: Supplementary file 2 — World Health Organization Trial Registration Data Set. (DOCX 19 kb) [file 13063_2018_3157_MOESM2_ESM.docx]

Table 1 World Health Organization Trial Registration Data Set

| **Data category** | **Information** |
| --- | --- |
| Primary Registry and Trial Identifying Number | German Clinical Trials Register (DRKS), DRKS00014685 |
| Date of Registration in Primary Registry |  |
| Source(s) of Monetary or Material Support | Federal Ministry of Education and Research (BMBF) |
| Primary Sponsor | Clinic of Child and Adolescent Psychiatry, Centre of Psychosocial Medicine, University of  Heidelberg, Blumenstrasse 8, Heidelberg 69115, Germany |
| Contact for Public Queries | Prof. Dr. med. Michael Kaess; Michael.Kaess@med.uni-heidelberg.de |
| Contact for Scientific Queries | Prof. Dr. med. Michael Kaess; Michael.Kaess@med.uni-heidelberg.de |
| Public Title | Promoting Help-seeking using E-technology for Adolescents with Mental Health Problems: Study Protocol for a Randomized Controlled Trial within the ProHEAD Consortium |
| Scientific Title | Promoting Help-seeking using E-technology for Adolescents with Mental Health Problems: Study Protocol for a Randomized Controlled Trial within the ProHEAD Consortium |
| Countries of Recruitment | Germany |
| Health Condition(s) or Problem(s) Studied | Mental Health problems |
| Intervention(s) | Internet-based intervention to promote help-seeking in adolescents with mental health problems |
| Key Inclusion and Exclusion Criteria | Children and adolescents (C&A) in grades 6-13 (≥12 years of age) are included if they endorse any form of mental health problems, including: serious suicide thoughts or attempts in the past two weeks, a score above 19 points on the *Strengths and Difficulties Questionnaire* total score, or a score above the defined thresholds for one of its sub-scales: emotional symptoms (scores >6), conduct problems (scores >4), or hyperactivity/inattention (scores >6). Further, C&A will be included if they report the following: Body Mass Index (BMI) < 5th percentile (adjusted for age and gender) AND concurrent fear of weight gain OR daily binge eating OR daily vomiting OR current alcohol use disorder (*AUDIT*) OR a score above 9 on the *Patient Health Questionnaire-9*. |
| Study Type | Randomized, multi-center, active control group, parallel group design |
| Date of First Enrollment | 01.11.2018 |
| Sample Size | 1,500 |
| Recruitment Status | Recruitment planned |
| Primary Outcome(s) | Actual help-seeking in the mental healthcare system at the time of the one-year follow-up |
| Key Secondary Outcomes | Frequency of contacts to professional mental health care system, long-term symptom development |
| Ethics Review | Approved by the Ethics Committee of the Medical Faculty at the University of Heidelberg (S-086/2018) |

*BMBF* Bundesministerium für Bildung und Forschung [Federal Ministry of Education and Research]; *DRKS* Deutsches Register Klinischer Studien [German Clinical Trials Register]
